# A Novel MFS Transporter Gene *ChMfs1* Is Important for Hyphal Morphology, Conidiation, and Pathogenicity in *Colletotrichum higginsianum*

**DOI:** 10.3389/fmicb.2017.01953

**Published:** 2017-10-10

**Authors:** Liping Liu, Yaqin Yan, Junbin Huang, Tom Hsiang, Yangdou Wei, Yu Li, Jie Gao, Lu Zheng

**Affiliations:** ^1^Key Lab of Plant Pathology of Hubei Province, Huazhong Agricultural University, Wuhan, China; ^2^Laboratory of Plant Pathology, Department of Agronomy, Jilin Agricultural University, Changchun, China; ^3^School of Environmental Sciences, University of Guelph, Guelph, ON, Canada; ^4^Department of Biology, University of Saskatchewan, Saskatoon, SK, Canada

**Keywords:** *Colletotrichum higginsianum*, infection, intra-hyphal hyphae, major facilitator superfamily transporter, pathogenicity

## Abstract

*Colletotrichum higginsianum* is a widely distributed fungus attacking many cruciferous species. To investigate pathogenic mechanisms of the pathogen on the host *Arabidopsis thaliana*, we screened and obtained a virulence-deficient mutant Ch-1-T513 in a T-DNA insertion mutant library of *C. higginsianum.* The mutant Ch-1-T513 produced yellow colony centers with distorted multi-branching hyphal tips as well as producing few conidia. Heavily swollen hyphae in the mutant could be observed, and intra-hyphal hyphae were found to be formed in the balloon-shaped hyphae. The mutant failed to produce lesions on 12-day-old *Arabidopsis* seedlings, and invasive hyphae did not differentiate into large primary and thin secondary hyphae after appressorial formation on *Arabidopsis* leaves, but formed abundant bulbous hyphae in epidermal cells. Southern blot analysis showed Ch-1-T513 had double-site T-DNA integrations. The mutant had insertions upstream of genes for a major facilitator superfamily (MFS) transporter, *ChMfs1* and an aldo/keto reductase, *ChAkr*. Complementation experiments by transforming genomic sequences from a wild-type strain into the insertion mutant demonstrated that *ChMfs1* is involved in the Ch-1-T513 phenotype. The complementation strain C-ChMfs1-1 exhibited normal hyphal morphology, conidiation, and pathogenicity identical to the wild-type. The results demonstrate that *ChMfs1* is involved in intra-hyphal hyphae production, conidiation, and pathogenicity in *C. higginsianum.* To our knowledge, this is the first report of a MFS transporter gene in a phytopathogenic fungus associated with intra-hyphal hyphae formation, playing a key role in infection of its plant host.

## Introduction

*Colletotrichum higginsianum* as a hemibiotrophic fungal pathogen causes anthracnose disease on many cruciferous plants, including economically important species, *Brassica* spp. as well as *Arabidopsis thaliana* (O’Connell et al., 2004). To infect plants, anthracnose conidia typically produce germination hyphae that differentiate into melanized appressoria, which in turn produce penetration pegs and enter into plant cell. Once inside the plant cell, the fungus forms swollen biotrophic primary hyphae which proliferate by invading living epidermal cells. These necrotrophic secondary hyphae spread more quickly across host cells causing chlorosis and necrosis. *A. thaliana* is an important model host with a completely sequenced genome available and ease of genetic analysis as well as an extensive mutant collection (O’Connell et al., 2004). Thus, the *C. higginsianum*–*Arabidopsis* pathosystem is an attractive model system for studying molecular mechanisms of plant–microbe interactions (O’Connell et al., 2004; Huser et al., 2009).

In natural environments, microorganisms always produce toxic chemicals to compete with other organisms and are capable of tolerating toxicity of these natural chemicals. Microorganisms have active transport systems which actively secrete synthetic and natural toxic compounds into the external environment. The ATP-binding cassette (ABC) and the major facilitator superfamily (MFS) of transporters are the two families which play an important role in these transport processes. ABC transporters are known well as primary active transporter systems. They hydrolyze nucleotide triphosphates and utilize the ATP energy to mediate membrane transport (Higgins, 1992). MFS transporters are regarded as secondary active transport systems and are not able to hydrolyze ATP. The proton-motive force involving membrane potential and electrochemical proton gradient drives transport of compounds though membranes (Lewis, 1994).

The MFS is one of the two largest superfamilies of membrane transporters present in eukaryotes and prokaryotes, and has members which function as symporters, antiporters, or uniporters. In plant pathogens, the function of several MFS transporters has been characterized and identified. The MFS transporters, *Bcmfs1* from *Botrytis cinerea* and *MgMfs1* from *Mycosphaerella graminicola*, are required for protection against fungicides and natural toxic compounds (Hayashi et al., 2002; Roohparvar et al., 2007). In several species of plant pathogens, MFS transporters are involved in secretion of phytotoxins including the host-specific toxin HC-toxin in *Cochliobolus carbonum* (Pitkin et al., 1996), non-host-specific toxins cercosporin in *Cercospora kikuchii* (Callahan et al., 1999), and trichothecenes in *Fusarium* *sporotrichioides* (Alexander et al., 1999). There are functional differences of the MFS transporters in several fungal species. We previously obtained a virulence-deficient mutant Ch-1-T513 from a T-DNA insertion mutant library containing over 5000 mutants in *C. higginsianum* using a high-throughout pathogenicity assay on *Arabidopsis* leaves. In this study, we found that the mutant producing intra-hyphal hyphae during infection phases had insertions upstream of genes for a MFS transporter and an aldo/keto reductase. In separate complementation studies for these two genes corresponding to the insertions, we demonstrated that the MFS transporter named *ChMfs1* is responsible for the mutant Ch-1-T513 phenotype. This study demonstrates that *ChMfs1* is involved in pathogenicity and formation of intra-hyphal hyphae during infection phases of *C. higginsianum* and complements a novel function of MFS transporters.

## Materials and Methods

### Strains, Vectors, and Plants

The *C. higginsianum* strain, IMI349061 (**Table 1**), originating from diseased plants of *Brassica campestris*, is maintained at CABI Bioscience (Egham, Surrey, United Kingdom). The *Agrobacterium tumefaciens* strain EHA105 was used in fungal transformation as the T-DNA donor (Qin et al., 2011). *Agrobacterium* was grown at 28°C on Luria Bertani (LB) agar supplemented with 50 μg each of kanamycin, rifampicin, and streptomycin per milliliter. *Escherichia coli* competent cell DH5α was used for plasmid transformation.

**Table 1:** Strains used in this study.

Strain	Description	Reference
Ch-1	*Colletotrichum higginsianum* IMI349063	O’Connell et al., 2004
Ch-1-T513	ATMT mutant from Ch-1	This study
C-ChMfs1-1	*ChMfs1* complementation strain from Ch-1-T513	This study
C-ChAkr-1	*ChAkr* complementation strain from Ch-1-T513	This study
EHA105	*Agrobacterium tumefaciens* competent cell	Liu et al., 2013
DH5α	*Escherichia coli* competent cell	Liu et al., 2013

Bacteria harboring binary vector pTFCM including the hygromycin B phosphotransferase (*hph*) cassette were used for *C. higginsianum* transformation (Li et al., 2005). Plasmid pNeo3300III with the neomycin resistance cassette were used for gene complementation vector construction (Liu et al., 2013).

The host plant *A. thaliana* ecotype Col-0 was used for all inoculation experiments. *Arabidopsis* seeds were placed on a peat-based compost, and incubated in a growth chamber (65–80% relative humidity) with a 16-h in light (40 μmol m^-2^ s^-1^, 400–700 nm) at 23°C, and 8-h dark at 18°C. The soil was composed of a perlite:vermiculite:sphagnum (1:1:1) mixture (Peilei, Qingzhou, China) and irrigated with a mineral nutrient solution as needed (Liu et al., 2013).

### Molecular Analysis of Mutant Ch-1-T513

In previous work, *A. tumefaciens*-mediated transformation system was used to generate an insertional mutant library of *C. higginsianum* wild-type strain Ch-1. Among 5012 transformants, we obtained mutant Ch-1-T513 which produced an intra-hyphal hyphae. Thermal asymmetric interlaced-polymerase chain reaction (TAIL-PCR) and inverse PCR were used for amplifying sequences flanking T-DNA insertions (Qin et al., 2011; Liu et al., 2013). The PCR products were cloned into the pMD18-T vector (TaKaRa, Dalian, China) and sequenced. And then the flanking sequences were used in BLAST against the *C. higginsianum* genome database^1^.

### Functional Complementation of Mutant Ch-1-T513

To verify that the two T-DNA integration events detected in the mutant Ch-1-T513 were responsible for the observed auxotrophy and pathogenicity phenotypes, ATMT was used to complement the mutant separately with a vector harboring a wild-type copy of the corresponding gene, *ChMfs1* or *ChAkr*. The complementation vectors designated pNeo3300IIIChMfs1-C and pNeo3300IIIChAkr-C were constructed. The 3.8-kb PCR product contained a 2000-bp upstream sequence, a full-length *ChMfs1* gene, and a 900-bp downstream sequence, and was amplified from genomic DNA of wild-type strain Ch-1 using primers COMPsp-BamHI-513-MFS/COMPap-BamHI-513-MFS (**Table 2**) and enzyme TaKaRa LA Taq (5 U/μl) (TaKaRa, Dalian, China). The BamHI-digested PCR fragment was then ligated to the BamHI site in pCAMBIA3300III to obtain pNeo3300IIICh-Mfs1-C. The 1.28-kb PCR product, containing a full-length *ChAkr* gene coding region, was amplified from the Ch-1 genomic DNA using primers COMPsp-BamHI-513-Akr/COMPap-BamHI-513-Akr (**Table 2**), and cloned into the pMD18-T vector. The BamHI-digested PCR fragment was then ligated to the BamHI site in pCAMBIA3300III to obtain pNeo3300IIIChAkr-C. The two complementation vectors were separately transformed into *A. tumefaciens* EHA105 and then integrated into corresponding mutant Ch-1-T513. The ATMT protocol was modified from that of Liu et al. (2013). Since the mutant Ch-1-T513 did not grow on potato dextrose agar (PDA) amended with antibiotic G418 (Amresco, Solon, OH, United States), the neomycin resistance cassette was chosen as the selectable marker for the complementation transformation. Complementation strains were screened on PDA containing 80 μg/ml G418 (Liu et al., 2013). Target gene and hph fragments of *ChMfs1* or *ChAkr* complementation transformants were primarily investigated by PCR amplification using primers MFSSP/MFSAP (for *ChMfs1*) and AkrSP/AkrAP (for *ChAkr*) (**Table 2**), and then Southern blot and gene expression were used for further confirmation. The complemented strains were then analyzed for phenotype and pathogenicity.

**Table 2:** Primers used in this study.

Primer	Sequence (5′–3′)
COMPsp-BamHI-513-MFS	TTAGGATCCCTTTCCAGCAATTTCCAAACCTC
COMPap-BamHI-513-MFS	AAAGGATCCCAATCTCCACAAGACAAACCCTC
COMPsp-BamHI-513-Akr	TTAGGATCCATGGCGCCCCCTATCTGCAC
COMPap-BamHI-513-Akr	AAAGGATCCTTACGCCAACCAGTTGCC
MFSSP	CTTTCCAGCAATTTCCAAACCTC
MFSAP	TCGGCAATAATTCAACCCAGACC
AkrSP	ATCTCGCTCGACGCCCTC
AkrAP	TTACGCCAACCAGTTGCC
MFSF	TGCGAGAAGATAGCGTGGAA
MFSR	AACCGTGGCGATGATGGAT
ActinF	ATGCGCCCAGAGCTGTCTT
ActinR	TTAGAAGCACTTGCGGTGGAC

### Phenotypic Analysis

To analyze hyphal morphology, 7-day-old mycelial plugs (5 mm in diameter) were transferred onto fresh PDA plates, and then incubated at 25°C in dark conditions. Hyphae picked from edge and center of the colony on PDA were examined by light microscopy (Nikon, Tokyo, Japan). The extent of radial hyphal growth was measured after 7 days.

For conidiation, strains were cultured on PDA plates at 25°C in dark conditions for 7 days. Conidia were harvested from 9 cm diameter Petri dishes by suspending in 5 ml sterile distilled water per disc, and centrifuging at 3000 × *g* for 2 min. The solution was decanted, and then were counted with a hemocytometer under a light microscope.

### Pathogenicity Tests

Conidial suspensions (10^6^ spores/ml) were prepared from the wild-type and mutants, and 10 ml was sprayed onto foliage of 12-day-old *Arabidopsis* plants for each pot (7 cm in diameter). After enclosing plants inside plastic translucent bags lined with wet tissue paper to provide high humidity, inoculated plants were incubated at 25°C with 16-h photoperiod at 40 μmol m^-2^ s^-1^. Symptoms were evaluated and leave tissues were sampled for light microscopy at 6 dpi.

### Light and Electron Microscopy

To evaluate fungal infection, inoculated leaf tissues were cleared in methanol/chloroform/glacial acetic acid (6:3:1) solution. To calculate the number of acervulus formation on leaves, infected tissues were then stained with lactophenol-trypan blue and cleared in chloral hydrate (Takahara et al., 2012). All leaf samples were viewed by differential interference contrast microscopy.

For scanning electron microscopy (SEM, following Cao et al., 2011), strains were cultured on PDA and sterilized glass slides were inserted in the media at an oblique angle by sterilized tweezers. After 7 days, the glass slides were coated with mycelia and conidia. The fungal samples were fixed in phosphate-buffered 2.5% glutaricdialdehyde in a series of increasing ethanol concentrations (30, 50, 70, 85, 95, and 100%), and dried using isoamyl acetate as the intermediate fluid in Critical Point Dryer (Model 13200-AB, SPI SUPPLIES, West Chester, PA, United States). Samples were sputter-coated with gold palladium using Auto Fine Coater (Model JFC-1600, NTC, Japan), and the mycelia of strains were observed with a JSM-6390/LV SEM (NTC, Japan).

For transmission electron microscopy (TEM, following Cao et al., 2011), hyphae were grown on PDA medium overlaid with cellophane membranes at 25°C for 7 days, collected, and prefixed with phosphate-buffered 2.5% glutaraldehyde (pH 7.2) at 4°C for 6 h. After rinsing several times with phosphate buffer, leaf tissues were fixed with phosphate-buffered 1.5% osmium tetroxide (pH 7.2) at 4°C for 2 h. The sections were then dehydrated with ethanol and embedded in SPI-812. Ultrathin sections were stained with 2% uranyl acetate and lead citrate, and observed with a Hitachi H-7650 electron microscopy (Hitachi, Tokyo, Japan).

### Nucleic Acid Manipulation, Southern Blotting, and RT-PCR

**FIGURE 1:**
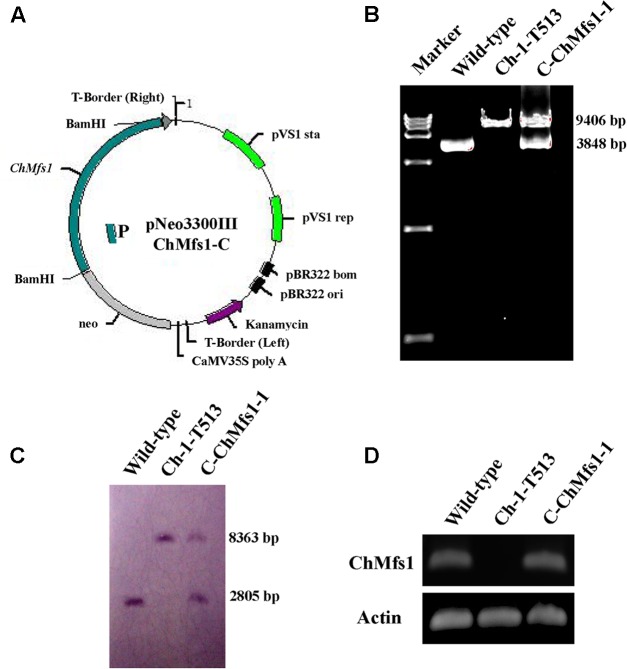
Complementation of *ChMfs1* gene in *C. higginsianum*. **(A)** Strategic map of gene complementation vector construction and sites for restriction enzymes at the *ChMfs1* genomic region. A bold line below the construct represents the sequence used as a probe (probe 1) in Southern blot analysis. **(B)** PCR analysis of wild-type Ch-1, T-DNA insertion mutant Ch-1-T513, and complementation strain C-ChMfs1-1. Marker in the image is DL15000. The 9406-bp band containing the sequences of *ChMfs1* gene and inserted *hph* cassette from T-DNA insertion vector pTFCM was amplified in the mutant Ch-1-T513 and complementation strain C-ChMfs1-1 while a 3848-bp fragment containing *ChMfs1* gene was obtained in the wild-type and C-ChMfs1-1. **(C)** Southern hybridization analysis of wild-type, T-DNA insertion mutant Ch-1-T513, and complementation strain C-ChMfs1-1. Genomic DNAs were digested with SacI and separated in 0.8% agarose gel. Blot was hybridized with the probe 1 amplified from genomic DNA of wild-type. **(D)** RT-PCR analysis of the transcription of wild-type Ch-1, T-DNA insertion mutant Ch-1-T513, and complementation strain C-ChMfs1-1. The 4-day old mycelia from PDA plates were collected and used for RNA isolation. Actin was used as the reference gene.

Hyphae were grown on PDA overlaid with cellophane membranes at 25°C, and genomic DNA was extracted using the CTAB method. For Southern blot analysis, 15 μg of DNA was completely digested for 24 h at 37°C with *Sac*I harboring only one recognition site in pTFCM. And then the digest was fractionated in a 0.8% agarose gel and mounted onto positively charged nylon membranes. The hygromycin resistance gene (*hph*) was excised from the pTFCM vector (Gong et al., 2007), and labeled with digoxigenin (DIG)-dUTP, using the PCR DIG Probe Synthesis Kit (Roche, Mannheim, Germany) following manufacturer’s instructions. Hybridization was detected using the DIG Luminescence Detection Kit (Roche, Mannheim, Germany). The nylon membrane was hybridized with probe P (**Figure 1A**). RNA isolation from 4-day-old mycelia was conducted using TRIzol Plus RNA Purification Kit (Invitrogen, Carlsbad, CA, United States), and then treated with DNase I (RNase Free) (Takara, Dalian, China). The first-strand cDNA was synthesized by RevertAid First-strand cDNA Synthesis Kit (Thermo Fisher Scientific Inc., Waltham, MA, United States). Expressions of *ChMfs1* gene in mutant strains and wild-type were examined by RT-PCR, and a 270-bp fragment was amplified with primers MFSF/MFSR (**Table 2**). The actin gene of *C. higginsianum* amplified with primers ActinF/ActinR was used as a reference gene (**Table 2**). PCR conditions were 34 cycles of 94°C for 30 s, 55°C for 30 s, and 72°C for 20 s and with a final extension at 72°C for 5 min. PCR reactions were run on a T-100 Thermal Cycler (Bio-Rad, United States).

### Bioinformatics

Primers were designed with Primer Premier (version 5.0^2^). Open reading frames were analyzed using FGENESH (Softberry Inc., Mount Kisco, NY, United States). Protein domain and motif predictions were performed with SMART software^3^. Top matching protein sequences from different organisms were downloaded from the GenBank database. Sequences were aligned using ClustalX (version 2.0^4^), and a phylogenetic tree was generated using the MEGA software (version 5.0^5^) with the Neighbor-joining algorithm. Bootstrap percentage values for branching support based on 1000 replicates are shown on the branches.

## Results

### Identification of ATMT Mutant Ch-1-T513

In previous work, for genome wide screening and identification of genes involved in pathogenicity in *C. higginsianum*, 5012 transformants were generated using the ATMT method and screened for the phenotypes and the ability to cause disease on *Arabidopsis* plants (Liu et al., 2013). Among transformants, one mutant, Ch-1-T513, showing abnormal hyphal morphology and conidial production was identified as a nonpathogenic strain, defective in pathogenesis on *Arabidopsis* leaves.

The integration pattern of the vector pTFCM in the mutant Ch-1-T513 was determined by Southern blotting, and two hybridizing bands were detected in genomic DNA from Ch-1-T513, indicating that Ch-1-T513 had double-site T-DNA integrations in the genome. To identify the location of the T-DNA insertion in the *C. higginsianum* genome, 2.1 and 1.8 kb genomic DNA flanking sequences were obtained using TAIL-PCR and inverse PCR, respectively. The flanking sequence of the ATMT mutant Ch-1-T513 was used to search the *C. higginsianum* genome database using BLASTx, pinpointing CH063_12120 and CH063_09290 as the targeted sequences of the T-DNA insertions. One T-DNA integration occurred 82 bp upstream of the CH063_12120 with significant similarity to a MFS transporter gene of *Coccidioides posadasii* (GenBank EFR28508.1; *e*-value = 7*e*-95) as well as other similarly annotated genes with low *e*-value matching, and the other T-DNA insertion was located 411 bp upstream of the CH063_09290 with significant similarity to an aldo/keto reductase gene of *Glomerella graminicola* (GenBank EFQ31134.1; *e*-value = 0) as well as other highly matching genes of similar annotation. Hence, these two T-DNA insertion genes were designated as *ChMfs1* and *ChAkr*.

### Insertion of T-DNA at the 82 bp Upstream of a MFS Transporter Gene Locus Is Responsible for the Ch-1-T513 Mutant Phenotype

To determine whether altered phenotypes and attenuated virulence in mutant Ch-1-T513 could be restored, we reintroduced a copy of the wild-type *ChMfs1*, by transforming mutant Ch-1-T513 with vector pNeo3300IIIChMfs1-C (**Figure 1A**). Potential complementation strains were confirmed by PCR, Southern blot, and gene expression analysis. In PCR analysis, the high molecular weight band of 9406 bp containing the sequences of *ChMfs1* gene and inserted *hph* cassette from T-DNA insertion vector pTFCM was amplified by *ChMfs1* gene amplification primers MFSSP/MFSAP in the mutant Ch-1-T513 and complementation strain C-ChMfs1-1, while a 3848-bp fragment containing the *ChMfs1* gene was obtained in the wild-type and C-ChMfs1-1 (**Figure 1B**). This revealed that inserted *hph* cassette was reliably detected in both transformants Ch-1-T513 and C-ChMfs1-1, meanwhile the *ChMfs1* gene was identified in wild-type and C-ChMfs1-1. In Southern blotting, a fragment from the *ChMfs1* gene was used as the probe (P) (**Figure 1A**), and a band of approximately 2805 bp was detected in wild-type and C-ChMfs1-1 (**Figure 1C**), similarly indicating that the sequence of *ChMfs1* was detected in the complementation strain C-ChMfs1-1. To examine the expression levels of *ChMfs1*, RT-PCR analysis of the mutant Ch-1-T513, complementation strain C-ChMfs1-1, and wild-type was conducted. In contrast with wild-type and C-ChMfs1-1, the *ChMfs1* gene expression was not detected in the mutant Ch-1-T513 (**Figure 1D**). The complementation transformant C-ChMfs1-1 exhibited a normal phenotype identical to the wild-type and recovered virulence on *Arabidopsis* leaves. The other corresponding gene (aldo/keto reductase gene) designated *ChAkr* was also reintroduced into the mutant Ch-1-T513 with vector pNeo3300IIIChAkr-C. We use the same methods to obtain the *ChAkr* complementation strain C-ChAkr-1. Complementation of the insertion at the locus of the aldo/keto reductase gene alone did not restore pathogenicity (data not shown).

### *ChMfs1* Is Highly Conserved in Phytopathogenic Fungi

**FIGURE 2:**
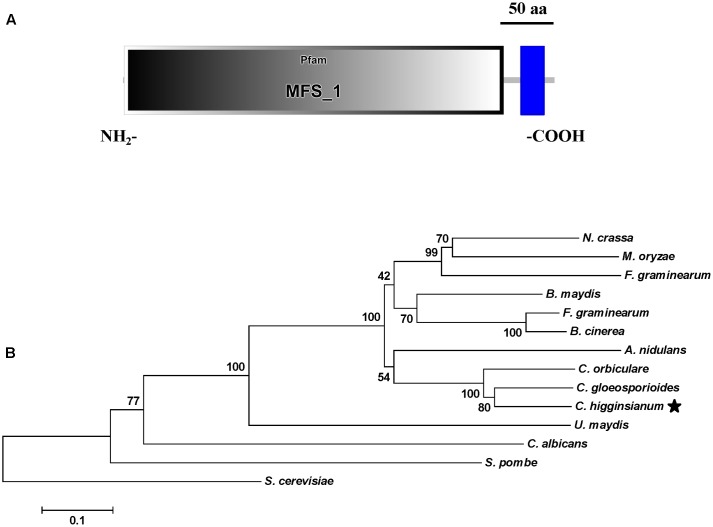
Functional domain identification and phylogenetic tree. **(A)** A conserved MFS_1 domain in *ChMfs1* was predicted using the SMART website. **(B)** Phylogenetic tree of putative *ChMfs1* identified in 14 fungal genomes. All of the *ChMfs1* proteins were downloaded from the NCBI database and their accession numbers are listed as follows: *Aspergillus nidulans*, XP_662973.1; *Bipolaris maydis*, XP_014083759.1; *B. cinerea*, XP_001560975.1; *Candida albicans*, KGU20518.1; *C. gloeosporioides*, XP_007272693.1; *C. higginsianum*, XP_018155340.1; *Colletotrichum orbiculare*, ENH82883.1; *Fusarium graminearum*, XP_011327285.1; *Magnaporthe oryzae*, XP_003712384.1; *Neurospora crassa*, XP_958596.1; *Saccharomyces cerevisiae*, AJU18388.1; *Schizosaccharomyces pombe*, NP_592802.1; *Sclerotinia sclerotiorum*, APA06707.1; *Ustilaginoidea virens*, KDB12461.1; and *Ustilago maydis*, XP_011392086.1. The numbers at branch nodes are bootstrap percentages out of 1000 replications.

Sequence analysis revealed that *ChMfs1* contains a MFS_1 domain at the N-terminus and transmembrane region toward the C-terminus (**Figure 2A**). A phylogenetic tree of MFS homolog proteins identified among various fungal genomes is shown in **Figure 2B**. The phylogenetic relationship of *ChMfs1* to other MFS proteins revealed that MFS proteins in filamentous fungi are separated from those of unicellular yeasts, with *ChMfs1* being most similar to that of *Colletotrichum gloeosporioides* and most distant from that of the basidiomycete *Ustilago maydis* and other yeasts (**Figure 2B**). The most distantly related sequences are still within 28% identity, so this indicates that *ChMfs1* proteins are well conserved in fungi.

### *ChMfs1* Is Required for Colony Morphology and Conidiation

**FIGURE 3:**
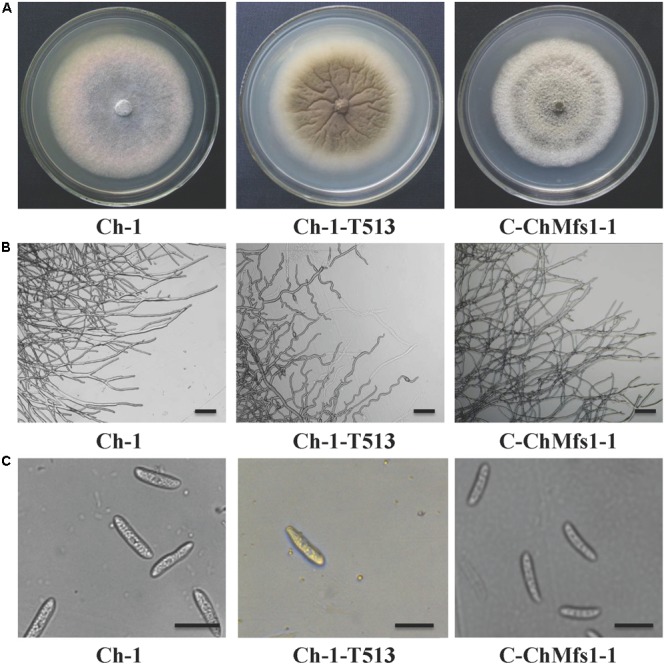
Hyphal and conidial morphology of *ChMfs1* mutant and complementation strain. **(A)** Colony morphology of wild-type Ch-1, T-DNA insertion mutant Ch-1-T513, and complementation strain C-ChMfs1-1 on PDA plates for 7 days. The mutant Ch-1-T513 produced yellow color in the center of colonies while the wild-type and C-ChMfs1-1 produced dark brown colonies. **(B)** Hyphae tips of the strains. Twisted hyphae were observed for the mutant Ch-1-T513 while normal hyphae were found in wild-type and C-ChMfs1-1. Hyphae tips picked from the edge of the colonies were examined by light microscopy. Scale bar = 10 μm. **(C)** Conidial morphology was not altered in the mutant Ch-1-T513. Conidia of wild-type, Ch-1-T513, and C-ChMfs1-1 were washed from PDA medium grown at 25°C in dark conditions for 7 days. Scale bar = 10 μm.

Growth and morphology of the wild-type, the T-DNA insertion mutant Ch-1-T513, and the complementation strain C-ChMfs1-1 were monitored. After growth on PDA for 7 days, the mutant Ch-1-T513 produced few aerial mycelium and became yellow in the colony centers, whereas the wild-type and C-ChMfs1-1 produced dark brown colonies with abundant white aerial mycelium (**Figure 3A**). However, the growth rate of the complementation strain C-ChMfs1-1 and the mutant was still significantly lower than that of the wild-type (**Table 3**). Examination under the microscope revealed that hyphal tips of the mutant Ch-1-T513 were twisted in contrast to a polar, linear hyphal growth of wild-type, and C-ChMfs1-1 which regained the ability to form normal hyphal morphology (**Figure 3B**). The conidiation of all tested strains was further examined on PDA plates post 7-day incubation. Although there were no morphological defects observed in conidia of the wild-type, Ch-1-T513, and C-ChMfs1-1, conidial abundance of C-ChMfs1-1 was recovered from the mutant Ch-1-T513 which was reduced by 84% of the wild-type (**Table 3** and **Figure 3C**). The results indicate that *ChMfs1* plays an important role in hyphal morphology and conidiation.

**Table 3:** Growth, conidiation, and formation of bulbous hyphae of *Colletotrichum higginsianum* mutants.

Strain	Vegetative	Conidiation	Formation of
	(mm/7 d)	(10^5^/plate)	bulbous hyphae (%)
Ch-1	58.5 ± 0.2	9.4 ± 1.1	0
Ch-1-T513	48.4 ± 0.1	1.5 ± 0.1	84.1 ± 5.3
C-ChMfs1-1	48.9 ± 0.3	7.5 ± 0.3	21.6 ± 2.4

### *ChMfs1* Is Involved in Producing Intra-hyphal Hyphae of *C. higginsianum*

**FIGURE 4:**
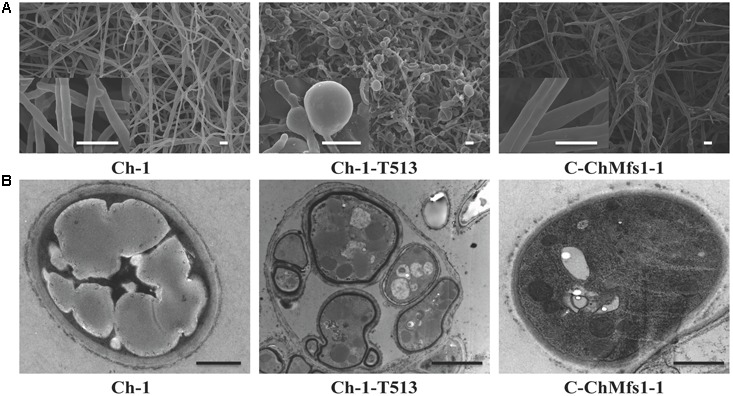
Scanning and transmission electron micrographs of hyphae in *ChMfs1* mutants. **(A)** Scanning electron micrographs of hyphae in wild-type Ch-1, T-DNA insertion mutant Ch-1-T513, and complementation strain C-ChMfs1-1. Swollen balloon-shaped hyphae were observed in the mutant Ch-1-T513 while complementation strain C-ChMfs1-1 recovered the hyphal morphology. Scale bar = 10 μm. **(B)** Transmission electron micrographs of hyphae in wild-type, Ch-1-T513, and C-ChMfs1-1. One hypha contained more than one intra-hyphal hypha in the mutant Ch-1-T513 in comparison to the wild-type and the complementation strain. The intra-hyphal strand of hypha has cell walls which clearly distinguish it from being a vacuole. Scale bar = 1 μm.

Heavily swollen hyphae visible under SEM in the mutant Ch-1-T513 almost fully reverted to wild-type in the complementation strain C-ChMfs1-1 (**Figure 4A**). Under TEM, intra-hyphal hyphae were seen to form in the balloon-shaped hyphae of the mutant Ch-1-T513. Moreover, single hyphal cells often contained more than one intra-hyphal hypha strand, where the cell walls were clearly delimited from that of the enclosing hyphae in the mutant Ch-1-T513 (**Figure 4B**). Intra-hyphal hyphae (84.1%) were frequently observed in the mutant Ch-1-T513 but were not found in the wild-type. Lower incidence of intra-hyphal hyphae (21.6%) was observed in the complementation strain C-ChMfs1-1 (**Table 3** and **Figure 4A**). This revealed that *ChMfs1* in the complementation strain could partially restore hyphal morphology. All of these observations suggest that *ChMfs1* is involved in producing intra-hyphal hyphae of *C. higginsianum.*

### *ChMfs1* Is Associated with Pathogenicity of *C. higginsianum*

**FIGURE 5:**
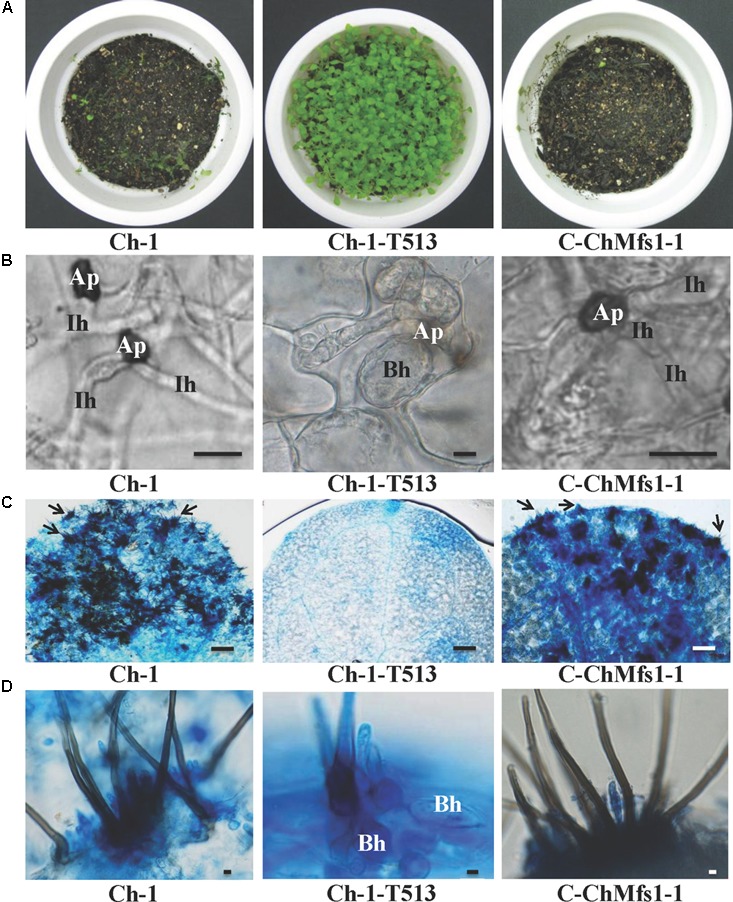
Deficiency in pathogenicity and acervulus formation of *ChMfs1* mutants on *Arabidopsis.* **(A)** Symptoms on 12-day-old seedlings at 6 dpi sprayed with conidial suspensions of wild-type Ch-1, T-DNA insertion mutant Ch-1-T513, and complementation strain C-ChMfs1-1. The wild-type and C-ChMfs1-1 caused completely collapsed and macerated lesions, whereas the mutant Ch-1-T513 caused no symptom. **(B)** Development of infection structures on *Arabidopsis* leaves at 4 dpi. Production of dark appressoria and infection hyphae on the surface of *Arabidopsis* leaves were found in wild-type and C-ChMfs1-1, but infection hyphae were not differentiated from appressoria in the mutant Ch-1-T513. Ap, appressoria; Ih, infection hyphae; Bh, balloon-shaped hyphae. Scale bar = 10 μm. **(C)** Acervulus formation on *Arabidopsis* leaves at 4 dpi. Few acervulus were found by mutant Ch-1-T513, but numerous acervulus were produced by the wild-type and complementation strain C-ChMfs1-1. Each arrow indicates an acervulus consisting of several melanized seta. Scale bar = 30 μm. **(D)** Balloon-shaped hyphae (Bh) formation on acervulus. Few balloon-shaped hyphae were found in the acervulus of the complementation strain C-ChMfs1-1. But numerous balloon-shaped hyphae were produced in acervulus by mutant Ch-1-T513. Scale bar = 5 μm.

After inoculation with conidial suspensions on 12-day-old *Arabidopsis* leaves, the mutant Ch-1-T513 failed to produce lesions, while the complementation strain C-ChMfs1-1 could cause necrotic lesions similar to the wild-type at 6 dpi (**Figure 5A**). Under microscopy, dark appressoria were observed to form on *Arabidopsis* leaves, but invasive hyphae of the mutant Ch-1-T513 did not differentiate into large primary and thin secondary hyphae, but formed abundant bulbous hyphae in epidermal cells and only three acervuli per leaf were produced (**Figures 5B,C**). However, conidia of C-ChMfs1-1 and the wild-type formed darkly melanized appressoria which produced penetration pegs into the epidermal cells and resulted in many acervuli (156 acervuli/leaf for C-ChMfs1-1 and 218 for the wild-type) (**Figures 5B,C**). The mutant Ch-1-T513 produced balloon-shaped hyphae in acervuli with few setae in lesions, whereas the wild-type and C-ChMfs1-1 produced normal acervuli with black setae in lesions (**Figure 5D**). The abnormal acervuli could not produce enough conidia to let the mutant Ch-1-T513 enter into infection cycle again. Therefore, we concluded that *ChMfs1* was involved in the formation of invasive hyphae after penetration and pathogenicity of *C. higginsianum*.

## Discussion

In this study, we obtained an avirulent mutant Ch-1-T513 by ATMT in *C. higginsianum* which showed intra-hyphal hyphae. The mutant had double-site T-DNA integrations with insertions upstream of genes for a MFS transporter (*ChMfs1*) and an aldo/keto reductase (*ChAkr*). Complementation experiments using the wild-type sequences corresponding to the two T-DNA insertion genes showed that *ChMfs1* alone is responsible for the phenotype of mutant Ch-1-T513. In human or animal pathogenic yeasts of *Candida* genus, a number of MFS transporters are well reported to be involved in host infection and virulence (Costa et al., 2014; Shah et al., 2014; Santos et al., 2017). However, intra-hyphal hyphae were not found in MFS transporter deletion mutants. A few MFS transporters from filamentous fungi also can act as virulence factors. This was reported for the HC toxin transporter from *C. carbonum*, the cercosporin transporter from *C. kikuchii* and the trichothecene transporter from *F. sporotrichioides* (Alexander et al., 1999; Callahan et al., 1999; Pitkin and Mullon, 1999). Among them, MFS transporters for secreting HC toxin or trichothecenes are located in a gene cluster that also encode enzymes required for biosynthesis of these toxins. However, these MFS proteins mentioned above have low level of homology to *ChMfs1* in *C. higginsianum* (with identities up to 31%), suggesting that there are significantly different domains between *ChMfs1* and these reported MFS proteins from *C. kikuchii*, *C. carbonum*, and *F. sporotrichioides*. Hence, *ChMfs1* in *C. higginsianum* is the first reported to be involved in pathogenicity and producing intra-hyphal hyphae. We believe that abnormal hyphae in the mutant Ch-1-T513 might be a key factor affecting virulence of *C. higginsianum.*

Intra-hyphal strands of hyphae refer to an interesting phenomenon where viable hyphae grow within dead or declining hypha cells, and this enables fungi to adapt to adverse conditions (Lane and Garrison, 1970). The formation of intra-hyphal hyphae is widely observed in many plant pathogenic fungi such as *Ceratocystis dryocoetidis* (Kendrick and Molnar, 1965), *Monilinia fructigena* (Calonge, 1968), *Verticillium albo-atrum* (Brown and Wyllie, 1970), *V. dahliae* (Wright, 1970), *Alternaria kikuchiana* (Ishizaki et al., 1976), *Botryosphaeria dothidea* (Kim et al., 2001), *Ustilaginoidea virens* (Kim and Park, 2007), and *Elsinoë fawcettii* (Kim and Hyun, 2007). Several impact factors had been reported to have the potential to induce or be associated with the formation of intra-hyphal hyphae in fungi. These include anaerobic cultural conditions (Miller and Anderson, 1961), stress from toxins and antibiotics (Lowry and Sussman, 1966; Ishizaki et al., 1976), injury and aging of mycelia (Chan and Stephen, 1967; Carbonell, 1969), fluidity of cultural media (Kim and Hyun, 2007), and mutation or deletion of genes associated with hyphal growth (Horiuchi et al., 1999; Kim et al., 2004; Takeshita et al., 2006; Martín-Urdíroz et al., 2008; Kim et al., 2009). In this study, formation of intra-hyphal hyphae was observed in the *ChMfs1* responding mutant Ch-1-T513 of *C. higginsianum*. We hypothesized that destruction of this key MFS transporter might result in deficiency of transport of toxic compounds, and abundant toxic compounds accumulated in the fungal cells. And then intra-hyphal hyphae were generated to resist adverse environment. This abnormal mycelial structure also could affect formation of infection structures. This is the first report of a MFS transporter gene associated with such structure formation, and also involved in reduced sporulation and inhibited formation of normal acervuli. These abnormal characteristics are similar to those of found with PDA cultures of the Δ*CgChsV* mutant strain of *C. graminicola* (Werner et al., 2007).

Formation of intra-hyphal hyphae in different fungi has been reported to have different origins. In *M. fructigena*, intra-hyphal hyphae originated from a zone between the cell membrane and the cell wall within a normal hyphal cell next to a degenerated hyphal cell (Calonge, 1968). These were found to originate from septa of existing hyphae in *C. dryocoetidis* and *V. albo-atrum* (Kendrick and Molnar, 1965; Brown and Wyllie, 1970). In *A. kikuchiana*, intra-hyphal hyphae formation was observed to be involved in the cell wall of parental hyphae, which expanded inward to divide the cytoplasm of the parental hyphal cells into several parts (Ishizaki et al., 1976). In this study, we found that formation of intra-hyphal hyphae is involved in pathogenicity and indentation of the hyphal cell wall at the penetration site of infected hyphae, indicating that mechanical force might be associated with the infection process.

Fungicides play an important role in modern agriculture by protecting crops against yield loss. Our results indicate the MFS transporter gene *ChMfs1* is a significant virulence factor affecting the infection of *C. higginsianum* on cruciferous plants. In future work, this virulence factor as a molecular target of *C. higginsianum* or even *Colletotrichum* spp. could be used for fungicide or inhibitor design in anthracnose disease management.

## Conclusion

In summary, the MFS transporter gene *ChMfs1* is important for hyphal morphology, conidation, and pathogenicity in *C. higginsianum*. Future studies to elucidate molecular mechanisms responsible for the formation of intra-hyphal hyphae in Ch-1-T513 are needed, and such information may lead to development of novel strategies to manage diseases caused by *C. higginsianum* and other *Colletotrichum* species.

## Author Contributions

Designed the experiments: LL, LZ, JH, and YW. Performed the experiments: LL and YY. Analyzed the experiment data: LL, LZ, YL, and JG. Contributed reagents/materials/analysis tools: LL and LZ. Wrote the paper: LL, LZ, and TH. All authors have read and approved the final manuscript.

## Conflict of Interest Statement

The authors declare that the research was conducted in the absence of any commercial or financial relationships that could be construed as a potential conflict of interest.

## Footnotes

1 http://genome.jgi.doe.gov/Colhi1/Colhi1.home.html

2 http://www.premierbiosoft.com/primerdesign/

3 http://smart.embl-heidelberg.de/

4 http://www.clustal.org/clustal2/

5 http://www.megasoftware.net/index.php
